# Implications of Lymphocyte Anergy to Glycolipids in Multiple Sclerosis (MS): iNKT Cells May Mediate the MS Infectious Trigger

**DOI:** 10.4172/2155-9899.1000144

**Published:** 2013-05-27

**Authors:** Edward L Hogan, Maria Podbielska, Joan O’Keeffe

**Affiliations:** 1 Georgia Regents University, Institute of Molecular Medicine and Genetics, Department of Neurology, 1120 15^th^ Street, Augusta, 30912-2620 GA, USA; 2 National University of Ireland Galway, Department of Microbiology, University Road, Galway, Ireland; 3 Medical University of South Carolina, Department of Neurosciences, 173 Ashley Avenue, Charleston, SC 29401, USA; 4 Ludwik Hirszfeld Institute of Immunology & Experimental Therapy, Polish Academy of Sciences, Laboratory of Signaling Proteins, R. Weigla Street 12, 53-114 Wrocław, Poland; 5 Department of Life and Physical Sciences, School of Science, Galway-Mayo Institute of Technology, Galway, Ireland

**Keywords:** Acetylated galactosylceramides, Anergy, iNKT cells, Glycosphingolipids, Multiple sclerosis

## Abstract

Immunogenic lipids may play key roles in host defenses against infection and in generating autoimmune inflammation and organ-specific damage. In multiple sclerosis (MS) there are unequivocal autoimmune features and vulnerability to aggravation or induction by microbial or viral infection. We have found glycolipid-driven anergy of circulating lymphocytes in MS indicating that this immune response is affected in MS and the robust effects of iNKT activation with potent cellular and cytokine activities emphasizes its potential importance. Diverse glycolipids including the endogenous myelin acetylated-galactosylceramides (AcGalCer) can drive activation that could be critical to the inflammatory demyelination in the central nervous system and clinical consequences. The iNKT cells and their invariant or iTCR (Vα24Jα18Vβ11) receptor an innate defense–a discrete immune arm that is separate from peptide-driven acquired immune responses. This offers new possibilities for insight including a likelihood that the pattern recognition of exogenous microbial and myelin immunogens can overlap and cross-react especially in an inflammatory milieu.

## Introduction

A relatively unexplored frontier in multiple sclerosis (MS) research is the role of immunogenic lipids and particularly complex glycolipids (GLs) and sphingolipids in disease mechanisms. Unlike peptides that reside internally, complex lipophilic molecules are on the microbial surface and first encounter host immune defenses in the course of infection. Hence, they qualify as possible “original antigens” igniting immune responses that can lead in the susceptible host to breaking tolerance and enabling autoimmune reactivity, inflammation, demyelination and symptomatic MS. The detailed mechanism of the development of intolerance is problematic but molecular over-lapping implicit in such suggestions as molecular mimicry [1] or polyspecificity [2] is a plausible means by which an ordinarily host-protective defensive immune response to infection (or an endogenous danger signal) is transformed into self-destructive attack. The slow evolution of research pursuing lipids as driving antigens in MS stems from their hydrophobic or partially hydrophobic (i.e. amphipathic) nature with solubility considerations that complicate assay and kinetic measurements because the limited aqueous solubility and molecular behavior of lipids affects antigen presentation as well as binding reproducibility and sensitivity.

## Lipid Antigens as a Target for Autoimmune Attack in MS

**Figure 1:**
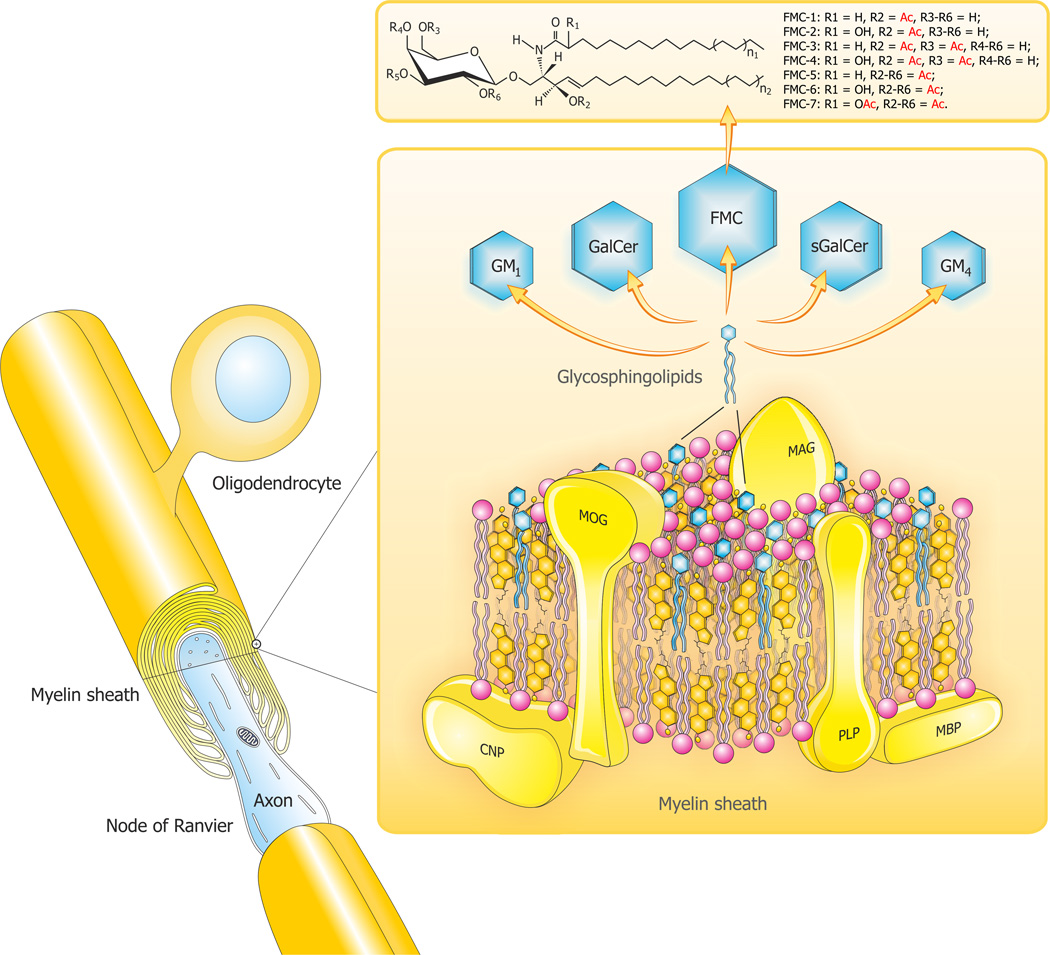
A composite diagram summarizing features of CNS myelin: i). architecture of CNS myelin; ii) molecular composition of CNS myelin (three-dimensional view) and; iii.) the unique sphingosine 3-*O*-acetylated-GalCer glycolipid series. The myelin is a multi-layer membrane formed by oligodendrocytes, containing a high lipid (80%) to protein (20%) ratio and an asymmetric distribution of lipids (PLP: Proteolipid protein; MBP: Myelin basic protein; MOG: Myelin oligodendrocyte glycoprotein; MAG: Myelin-associated glycoprotein; CNP: 2’3’-cyclic-nucleotide 3’-phospodiesterase). The diagram depicts a hypothetical arrangement according to molar compositions of complex lipids (cholesterol, phospholipids and glycolipids) and the most abundant proteins (PLP, MBP) in the CNS myelin bilayer. The relative constancy of molar proportions of the three lipid classes: cholesterol (C): phospholipids (PLs): galactocerebrosides (GalCer) distributed in bilayers is C: PLs: GalCer=2:2:1. Proteins abbreviated as above are marked in yellow and the comprising lipids are as follows: cholesterol in orange, phospholipids in pink and the glycosphingolipids (FMC: Fast migrating cerebrosides; GalCer: Galactosylceramide; GM1: Mono-sialoganglioside; GM4: Sialosyl-galactosylceramide; sGalCer: sulfatide) in blue. Structures of myelin acetyl-cerebrosides (FMCs) are shown at the top. Seven GalCer derivatives, have been characterized in vertebrate brain myelin including human. FMC-1/-2 are 3-*O*- acetyl-sphingosine derivatives, FMC-3/-4 add 6-*O*-acetyl-galactose, and the complex FMC-5/-6/-7 are 2,3,4,6-tetra-*O*-acetyl-GalCers. Penta- and hexa-acetylated complex FMCs are hydrophobic lipids capable of affecting myelin membrane curvature, lipid interactions, and immune reactivity. Adapted and modified from Podbielska et al. [5].

The target organ of MS is mainly myelin though other CNS cells including neurons and their axons, astrocytes, microglia, endothelial cells or pericytes may also be affected. Our interest in the lipids of myelin and their established and potential roles as immunogens and bioactive mediators in the mechanisms of the inflammatory demyelination of MS has been described [3–6] and will here be only briefly summarized. Myelin is a unique multilayered membrane investing axons and facilitating saltatory conduction. Myelin is highly concentrated in complex lipids and sterols with an asymmetric arrangement of GLs and also phosphatidylethanolamine in the outer membrane [7] while phosphatidylserine predominates on the cytoplasmic face [8]. The main myelin lipids and other myelin constituents are depicted in Figure 1. Galactosylceramides (GalCer) have long been known to bind to CNS-derived immunoglobulins [9]. Other GalCer derivatives are sulfated, sialylated and acetylated; specific compounds being sulfatide [10], GM4 ganglioside (sialosyl-galactosylceramide) [11], GM1 monosialganglioside and the acetyl-galactosylceramides (AcGalCer) [12–14]. All of these are potent immunogens as well.

MS is the most common demyelinating disease of man and seems to be mediated by auto-reactive CNS-specific CD4^+^ or other T cells with a role for B cells too. The demyelination is largely inflammatory in nature and the pathogenic events are believed to follow a sequence for T cells of initial priming, activation in the periphery by cytokines or other means, migration across the endothelial cell and blood-brain barrier, and re-activation during CNS invasion. There have been many reports describing changes in lipids in MS plaque lesions and in normal-appearing white matter (NAWM). The main or most compelling ones will be briefly reviewed in the following with particular attention to gangliosides, sulfatides, phospholipids, and AcGalCer. In the past, AcGalCers have also been operationally called fast-migrating cerebrosides (FMCs) reflecting the TLC behavior that enabled resolution from gluco- and galactosylceramides and purification for structural characterization [15]. In MS brain total lipid content is decreased and myelin-enriched lipids decreased or absent. Cholesterol is decreased in both NAWM and normal appearing gray matter (NAGM) in MS brain [16]. Other tantalizing alterations in levels or molecular state (e.g. lipid oxidation or degradation) have been described (see below). They are interesting because of the effect of the molecular structural alterations upon immunogenicity.

Gangliosides are abundant in brain and associated with neuronal surface or axolemma while GM1 is increased in concentration in myelin. Their concentration is reduced in MS and composition altered such that plaques are devoid of GM4; GM1 and GM2 are reduced, and; GD2 & GD3 are elevated as are GQ1 and other polysialo-gangliosides [17]. Only minor changes in lipid concentration have been observed in MS white matter [18].

Sulfatides are reduced in MS plaques and less so in WM with a sparing of the hydroxylated species especially hC24:0 sulfatide [19].

Phospholipids (PLs) are of interest in MS because anti-PL antibodies reportedly increase though not all workers found this [20,21]. Intriguing relevant additional observations include an elevation of choline in NAWM that evolves into plaque [22,23], and an increase in oxidized phosphatidylcholine in MS but not control brain [24].

## Anti-glycolipid Reactivity in MS

Autoantibodies occur in autoimmune disorders, may be proportional in titer to disease severity and could be valuable markers for diagnosis, classification, assessment of disease activity and future course [25]. Roles for pathogenic antibodies are the main issue but protective and repair functions have been observed and at times the nature of the binding could not be determined. Antibody elevations in MS CSF affect mainly the IgG1 isotype; occasionally the antibody is IgG3.

For decades, effort has been directed mainly to the study of the immune response to proteins found in myelin [26–31] while the role of myelin lipids has not been strictly defined [4] until more recently when the determination of anti-lipid specificity has become an important avenue of MS research.

Of the GLs, GalCer, which accounts for 32% of CNS myelin lipid content, was mainly targeted for an auto-antibody immune response and recently anti-GalCer antibodies with demyelinating potential [32,33] have been found in MS, particularly in RRMS and not in healthy controls [34]. Since these antibodies are not detected to any extent in patients with early-stage MS in the clinically isolated syndrome (CIS), it would be useful to monitor CIS patients for these anti-GalCer antibodies in order to predict conversion to clinically definite MS. Antibodies in MS to other GLs such as gangliosides [35–37], sulfatides [38,39], and PLs [40] have been also described, and might be associated with disease progression. We have attempted to define the specificity of antibodies for complex lipids [14]. Consistent with reports of MS CSF antibodies determined by lipid array analysis the findings suggest that the oligoclonal IgGs in MS brain and CSF react with myelin lipids that are released during myelin breakdown during demyelination with antibodies that are particularly reactive with sulfatide, sphingomyelin, several PLs and oxidized PLs, and oxidized sterols [41,42]. Overall, we believe that the increased and oligoclonal immunoglobulin in MS CSF is directed at the tissue inflammatory response encompassing a range of complex lipid antigens that are mostly myelin lipids and with degradation products including bioactive signal molecules [6]. MS patients with higher IgG levels and polyclonality (i.e. more oligoclonal IgGs) are reported to have a poorer prognosis [43,44]; thus greater inflammatory response and greater autoimmune reactivity is ominous. Intrathecal IgM synthesis has also been associated with rapid MS progression and is associated with lipid immunogenicity particularly of phosphatidylcholine [45].

## Glycolipid Ligands for iNKT Cells

**Figure 2:**
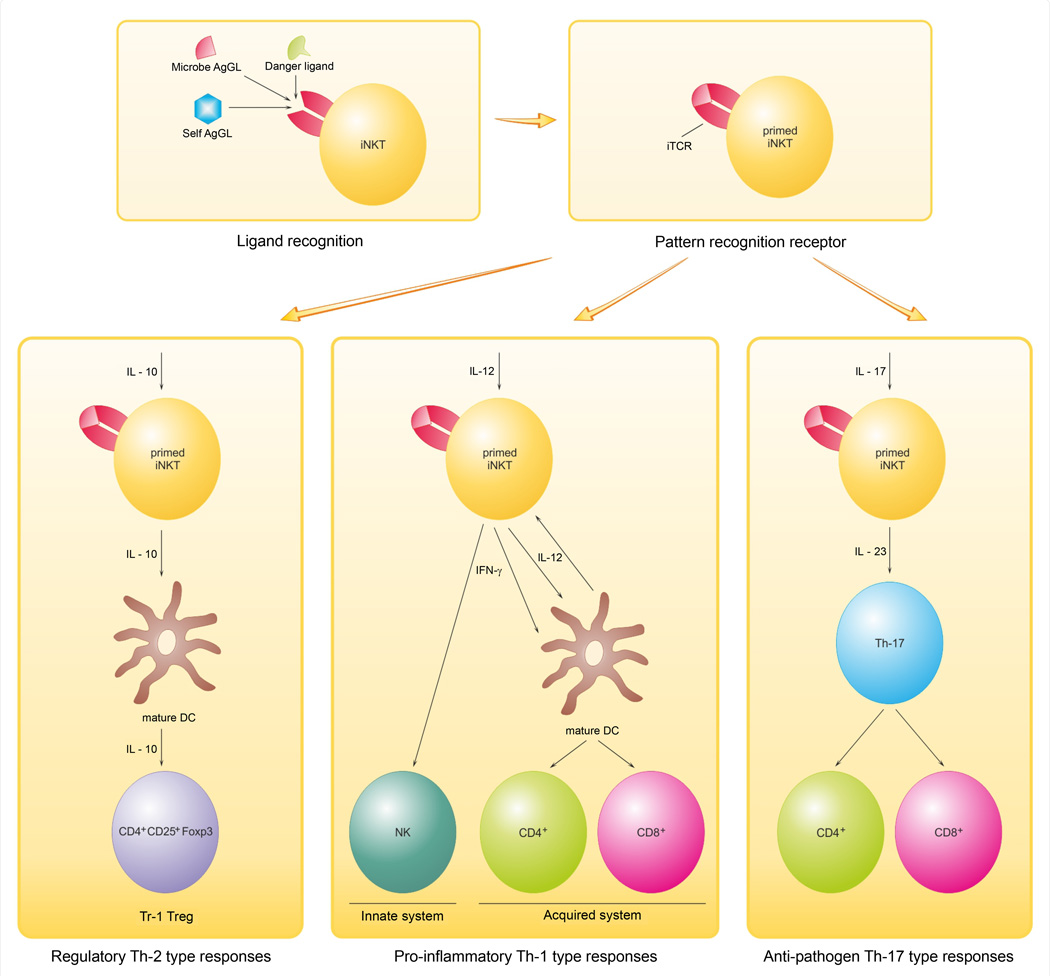
iNKT cell-mediated responses in the immune system. Several different antigenic GLs: microbial GLs; danger ligands as from cellular oxidation; and self antigens (Ags; in MS case they are myelin GLs) compete for binding to iTCR. The iTCR is a PRR that are ’pattern recognition receptors’ of the innate immune system. This recognition allows immune responses to different lipids–e.g. GLs, PLs and these can allow immune responses to microbial GLs (usually with high affinity) to overlap with those to self Ags (weaker affinity as a rule). The iNKT is 1) primed by the GL/iTCR/Cd1d receptor-located interaction or interface, and then 2) respond in one of several ways according to the environment, mainly cytokines-dependent. This affects control of diverse outcomes: i) pro-inflammatory (Th-1 type responses), ii) regulatory (Th-2 type responses) or iii) anti-pathogen (Th-17 type responses). Initial and subsequent interaction with IL-12 leads to iNKT pro-inflammatory functions. IL-12-activated iNKT mediate adjuvant activity by their production of IFN-γ, which in turn activates both innate and acquired immune systems. In contrast, interaction with an IL-10-driven response generates regulatory Tr1 Tregs (e.g. CD4^+^CD25^+^Foxp3) that are charged with development and maintaining tolerance. In addition to their roles in autoimmunity and tolerance, iNKT cells have also anti-pathogen activity triggered by IL-17 and IL-23. Adapted and modified from Taniguchi et al. [46].

Several types of regulatory cells including CD8^+^ T cells, B cells and NKT cells participate in controlling pathological autoimmunity [4]. We have focused upon NKT cells because the invariant NKT cells are GL-reactive and constitute a distinct and discrete arm of the immune system that is separate from conventional peptide-binding T cells [46]. The invariant NKT (iNKT or type I NKT) cells employ a single species of TCR encoded by Vα24Jα18 α-chain gene segments in humans [47]. This iTCR binds mainly GL antigens and requires for lipid presentation CD1d; a non-classical monomorphic MHC-related antigen presenting cell (APC) surface protein [47]. The breadth of molecules reacting with the iNKT receptor (iTCR) categorizes the iTCR as an innate pathogen recognition receptor (PRR). The crystal structure of the complex formed by the potent ligand α-galactosylceramide (α-GalCer), human CD1d and the iTCR (Vα24Vβ11) has been characterized and shows binding sites for both the GL ligand and CD1d on the iTCR α-chain [48]. Both the iTCR and CD1d are conserved emphasizing the importance of NKTs in man. The binding reactivity of the invariant TCR encompasses many GLs [49] that prime the iNKT cell for activation mainly by cytokines including IL-12, IL-10 and IL-17 for diverse activation. Prominent immune responses include the following: (i.) Th1-biased inflammation, (ii.) regulatory T cell maturation, and (iii.) pathogen defense [46] and we have depicted a scheme (Figure 2) to outline the GL ligand/iTCR binding and subsequent T cell priming and then activation in different ways as determined and mediated by the local environment. Conserved cytokine activation and diversification in MS for these and related cytokines has long been recognized [50]. INKT cells are cytokine-rich, potent and versatile, and the iNKT cell interface with innate GL conformational signals for infections or for intrinsic dangers is a transformational bridge able to elicit specific cytokine messaging by IL-1, TNF-α, IL-12, IL-17 and others en route to specific responses by the acquired immune system. INKT cells produce large amounts of IFN-γ and IL-4 upon activation particularly by α-GalCer [51,52] and have diverse effects *in vivo* including regulation of autoimmunity [53].

**Figure 3:**
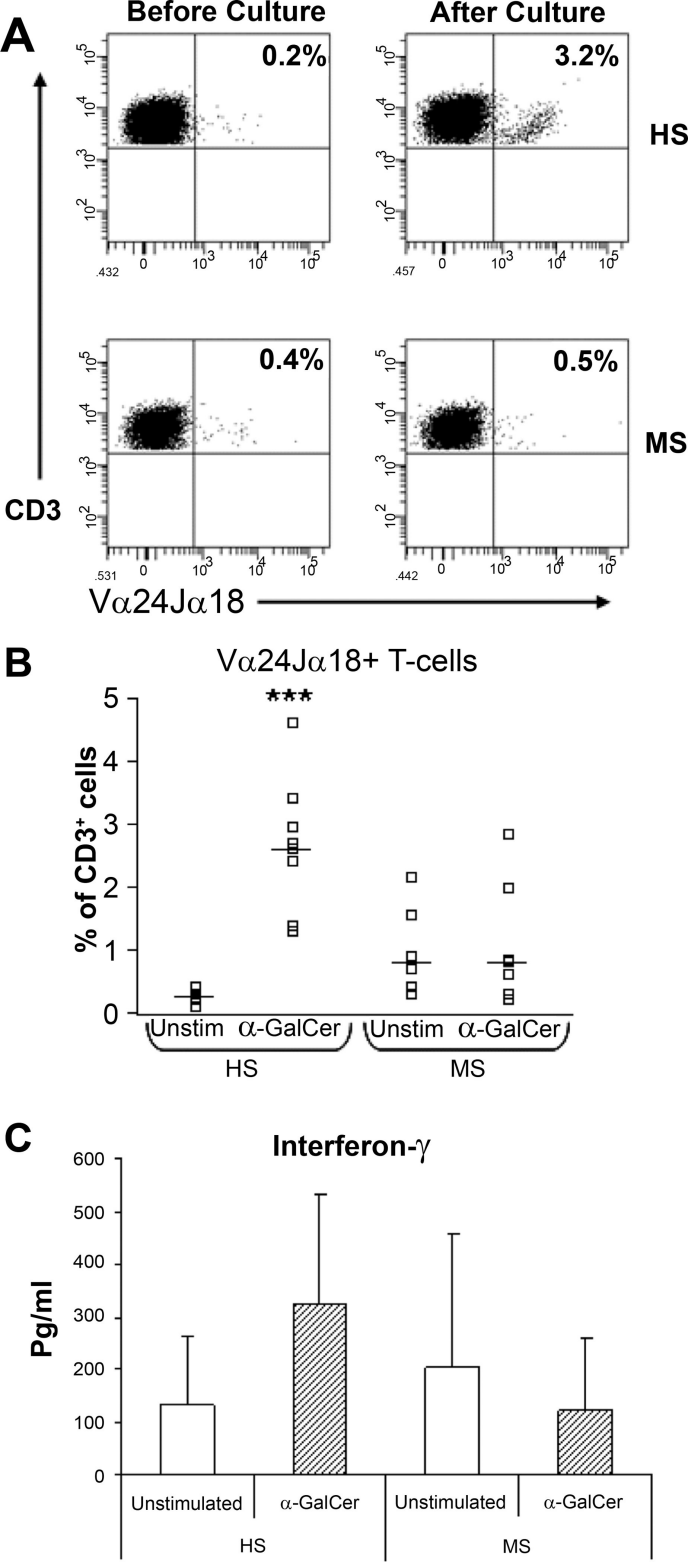
NKT-cell responses to α-GalCer are impaired in MS. (A) Representative flow cytometric profiles of Vα24Jα18+ T-cells (NKT-cells) from one healthy subject (HS) and one MS patient. The frequency of NKT-cells before and after culture with α-GalCer is shown in the upper-right hand quadrants in each plot. (B) Percentages of NKT-cells in unstimulated (medium alone) and α-GalCer stimulated cultures in 8 healthy subjects and 7 MS patients. Horizontal bars indicate median levels. (C) IFN-γ production in unstimulated PBMC cultures or in α-GalCer stimulated cultures in HS and MS patients. Data shows mean levels and error bars show the standard deviation. Significance values comparing HS to MS patients are shown by ***, P<0.001. From O’Keeffe et al. [57].

We examined iNKT cell function (Figure 3) in circulating peripheral blood cells in MS patients.

In recent years an important role for T-cells that bear natural killer (NK) receptors has been recognized in regulating autoimmune diseases like MS [54,55]. Included in this group are the invariant NKT-cells that express NK-cell surface receptors and a highly restricted T-cell receptor (TCR) repertoire, encoded by Vα24 and Jα18 genes in humans [46,47]. INKT and other innate immune cells like γδ T-cells [56] act as front-line immune regulatory cells [54]. Because these T-cells play important roles in regulating human autoimmune diseases, we quantified T-cells populations expressing the NKR CD56, CD161 and CD94 in the peripheral blood of MS patients, in healthy control subjects (HS) and in patients with other neurological diseases (OND) [57] and showed that populations of CD161^+^ T-cells and CD94^+^ T-cells were significantly decreased in MS patients with primary progressive disease and secondarily progressive disease respectively whereas CD56^+^ T-cell numbers were unchanged. In contrast NKT-cells expressing the invariant Vα24Jα18^+^ T-cell receptor identified by specific receptor antibody and CD1d-tetrameric PBS57-loaded complexes, were increased in MS patients compared with healthy subjects. Alterations in the proportions of NKR^+^ T-cells in MS may be clinically relevant since reduced numbers could insufficiently activate populations required for controlling disease activity: this has been shown for the functional activities of NKR^+^ T-cells in tumour immunity [58]. Importantly, the reductions in these NKR^+^ T cell numbers may reflect a decrease in immune inhibition with consequent progression of the neurodegenerative phase of MS. We also employed flow cytometry and cytokine assay to study the functional responses of the NKR^+^ T cells to stimulation with α-GalCer and to two myelin-derived GLs that are poly-acetylated derivatives of β-galactosylceramide designated as FMCs [59]. In healthy subjects, FMC stimulation of peripheral blood cells significantly expanded iNKT-cells similar to α-GalCer and induced significant increases in Th1, Th2 and Th17 cytokines. Importantly, the GL response as measured by an expansion in cell number was specific to the iNKT-cell population: there were no increases in the frequencies of either NK cells or NKR^+^ T-cells (CD56^+^ T-cells, CD161^+^ T-cells and CD94^+^ T-cells) upon stimulation with any of the GLs tested. The results with MS patients were in striking contrast to healthy control subjects. INKT-cells from MS patients failed to respond to FMCs or to α-GalCer stimulation indicating an anergic response. We propose then that myelin-derived FMC GLs stimulate iNKT-cell responses *in vivo* and this is blocked in MS. Rendering iNKT-cells hyporesponsive to an endogenous GL is a novel insight into diseases manifesting aberrant iNKT-cell activation and consequently this finding of GL ligand-driven anergy in MS has substantial implications for MS. The loss of responsiveness or anergy was to the exogenous α-GalCer ligand [57] as well as to the endogenous polyacetylated-GalCers (FMCs) [59] that we had previously purified and characterized [14]. Furthermore the numbers of iNKT cells significantly expanded upon stimulation with α-GalCer and the FMCs accompanied by robust cytokine secretion in healthy control subjects [57,59]. These included cytokines associated with Th1 cells (IFN-γ), Th17 cells (IL-17, TNF-α) and both pro-inflammatory (IL-1β, IL-6, TNF-α) and anti-inflammatory responses (IL-10). IL-17 expression is upregulated and involved in the pathogenesis of MS in humans [60] and also in EAE [61].

Since α-GalCer ameliorates or prevents EAE [62,63] and another innate indicator, activation of γδ T cells by lipid antigens, rises in MS [64], implications for MS mechanisms are likely. The anergy that is consistent with previous iNKT cell usage, implicates innate iNKT immune reactivity and probably reflects saturation of the iTCR by GL antigen due to infection or to release of myelin GLs during MS demyelination. We think as outlined in Figure 2 that the rivalry at the receptor level of high-iTCR-affinity microbial GLs with weaker-binding myelin GLs initiates iNKT cell activation that depends upon the presence of cytokines mediating either damaging Th1-biased (Il-12 driven) or pathogen directed (IL-23 & IL-10 driven), or regulatory T cell (Tr-1 cells) processes [46]. The scheme admittedly simplifies the complexity of the cytokine and other influential controls but allows for plausible hypotheses for the transformation of an infection-dependent process to an autoimmune one with loss of tolerance for endogenous myelin biomarkers. It also provides a basis for examining the inflammation and other events underlying MS’s complicated pathogenesis. Our scheme for the sequences of GLs-reactivity and iNKT-mediated consequences that occur in MS CNS stresses competition for GL antigens that underlies the cross-reactivity that allows tolerance for self myelin lipid antigens to be broken and then for myelin-targeted inflammation directed by local environmental signals especially IL-12, -10, -23 and -17. Pursuing the GL and lipid-specific cells in MS will yield insights into lipid use in anti-microbial vaccines and whether CD1d-restricted T cell strategies can treat MS.

## The Glycolipid-iNKT Pathway Can Trigger Infection-related Autoimmune Disorder

The evidence the MS is triggered by infection is both clinical with an increased frequency of infection in conjunction with relapse [65–67] and laboratory-based demonstration of infectious agents [68,69] and supports an interpretation that infection precipitates or activates CNS inflammation in MS. Antibodies to many viruses and bacteria [70–75] and reactivity of lymphocytes [76–79] have been examined, and reactivity with measles, mumps, herpes (HSV-1), varicella (VZV), cytomegalovirus (CMV), and Epstein-Barr virus (EBV) [70–75] have been reported though titers and affinities for these have been low, and the binding specificity of the majority of intrathecal antibody in MS is not known. Taken together it appears clear that systemic infections may trigger a relapse or exacerbate existing symptoms of MS.

Molecular overlap and consequent cross-reactivity also occurs in several other immune-mediated inflammatory neurological disorders involving peripheral nervous system (PNS). In these disorders there is relatively strong experimental support for a role of GL and particularly the glycose moiety as immunogenic. Immune mechanisms play a crucial part in Guillain-Barré syndrome (GBS) pathogenesis. About two-thirds of GBS patients have symptoms of an infection in the 3 weeks before the onset of weakness. The most frequently identified infection is *Campylobacter jejuni*. Other specifically characterized types of infection related to GBS are cytomegalovirus, Epstein- Barr virus, *Mycoplasma pneumonia* and *Haemophilus influenza*. In about half of patients with GBS and the clinical variant syndromes in the forms of: (a.) acute motor axonal neuropathy [acute motor axonal neuropathy (AMAN) or (b.) acute motor and sensory axonal neuropathy (AMSAN)] and (c.) a cranial nerve variant of GBS, Miller Fisher syndrome (MFS) [80]. Serum antibodies have been observed in GBS and related disorders to various gangliosides that have been found in human peripheral nerves, including LM1, GM1, GM1b, GM2, GD1a, GalNAc-GD1a, GD1b, GD2, GD3, GT1a and GQ1b. Other antibodies bind to mixtures or complexes of different gangliosides instead of binding individual gangliosides. Interestingly, most of these antibodies are specific to defined subtypes of GBS. Antibodies to GM1, GM1b, GD1a and GalNAc-GD1a are associated with pure motor or axonal neuropathy (AMAN or AMSAN), whereas antibodies to GD3, GT1a and GQ1b are related to the MFS variant form of acute inflammatory GBS neuropathy affecting control of eye movement (ophthalmoplegia) [80]. In addition, GBS is more severe when antibodies against GM1 are found. Clinical examples of this are chronic inflammatory demyelinating polyneuropathy (CIDP) with anti-GM1 and LM1 antibodies [80] and multifocal motor neuropathy (MMN) with 33% manifesting IgM antibodies against GM1 and GM1a. Another PNS example is sensory neuropathy with antibodies to GD1b and disialogangliosides. *C. jejuni* isolates from patients with pure motor or axonal GBS frequently express a GM1-like and GD1a-like glyco-epitopes on liposaccharides (LPS), whereas those isolated from patients with ophthalmoplegia or MFS usually express a GD3-like, GT1a-like, or GD1c-like LPS. Antibodies in these patients are usually cross-reactive, and recognize LPS as well as gangliosides or ganglioside complexes strongly suggesting that molecular mimicry of PNS myelin gangliosides and microbial LPS is a mechanism of the autoimmune neuropathy [81].

## A Future Perspective

Identifying the antigens that play roles in the pathogenesis of maladaptive MS autoimmunity is crucial to understanding MS. Within a context of genetic and environmental factors that are complex and not fully understood but with key roles for both determinants, microbial triggering of autoimmunity occurs when the usual immune defense against pathogenic microbes with concurrent tolerance for myelin antigens loses non-self/self discrimination and allows myelin antigens to react with host immune elements and produce myelin-specific inflammation and damage. The conformational plasticity of complex lipids and especially GLs suffices for the range of an innate pattern recognition receptor the iTCR, Vα24Jα18Vβ11, and allows overlapping molecular diversity with consequent loss of tolerance leading to maladaptive organ-specific inflammation affecting CNS and particularly myelin. Our studies of the GL-reactive separate immune arm mediated by iNKT cells that revealed anergy of circulating lymphocytes in MS to the potent exogenous stimulant α-GalCer now extend to endogenous myelin lipid antigens; the acetyl-β-GalCer series. The indication that utilization of the iNKT pathway is a feature of the infection-MS transition linking infection and autoimmune disorder not only offers novel insight into MS pathogenesis but may also have more fundamental importance indicating a mechanism for organ GLs to initiate induction of numerous other organ-targeted autoimmune disorders such as myasthenia gravis and rheumatoid arthritis. To us the prospect of gaining molecular insight into the mechanism of the infectious trigger adds welcome light to the darkness enveloping the enigmatic and disabling scourge MS.
